# Characterization of the complete mitochondrial genome of three-spined stickleback, *Gasterosteus aculeatus*

**DOI:** 10.1080/23802359.2018.1473732

**Published:** 2018-09-27

**Authors:** Jin-Qing Jiang, Qiu-Xia Wang, Jun-Wei Liu, Chang-Zhong Liu

**Affiliations:** College of Animal Science and Veterinary Medicine, Henan Institute of Science and Technology, Xinxiang, China

**Keywords:** Three-spined stickleback, *Gasterosteus aculeatus*, mitochondrial genome, phylogenetic analysis

## Abstract

In this study, the complete mitochondrial genome of three-spined stickleback, *Gasterosteus aculeatus,* was determined through sequencing of PCR fragments. The complete mitochondrial genome of *G. aculeatus* was 16,543 bp in length and encoded 13 protein-coding genes, 22 transfer RNA (tRNA) genes, and two ribosomal RNA genes. The overall nucleotide composition is: 27.0% A, 28.4% T, 27.4% C, and 17.2% G, with a total G + C content of 44.6%. By phylogenetic analysis using ML method, *G. aculeatus* showed the closest relationship with the blackspotted stickleback (*Gasterosteus wheatlandi*).

The three-spined stickleback (*Gasterosteus aculeatus*), as an important model in the study of adaptive evolution, is a species of fish in the family Gasterosteidae, which includes five genera and 15 other species. The ancestral anadromous populations of three-spined stickleback have repeatedly and independently colonized freshwater throughout the Northern Hemisphere (Taylor and McPhail 2000; Mäkinen et al. 2006). In this study, *Gasterosteus aculeatus* were collected on Bear Paw Lake, Alaska (61skated 149skatede Muscle tissues were preserved in 100% ethanol and stored at –40 °C until DNA extraction. Whole genomic DNA was extracted from a single sample using the commercial Animal Tissues Genomic DNA Extraction Kit (Solarbio, Beijing, China) following the manufacturer’s instructions, and then used as the template for polymerase chain reaction (PCR) amplifications. The quality of PCR products was assessed through electrophoresis in a 1% agarose gel. The isolated DNA was stored in the sequencing company (BGI Tech, Shenzhen, China). The amplified products were sequenced using the amplification primers. All sequencing was done by a commercial sequencing service (BGI Tech, Shenzhen, China).

The complete mitogenome of *Gasterosteus aculeatus* was obtained by the assembly of 12 overlapping sequences via the alignment of neighbouring fragments using SeqMan in the DNASTAR 7.1 (Burland 2000). Thirteen PCGs and two rRNA genes were inferred based on comparison with mitogenome sequence of *Gasterosteus wheatlandi* (GenBank accession: AB445129.1) combined with predictions through the MITOS web server (Bernt et al. 2013) using the vertebrate mitochondrial genetic code. The location and secondary structure of the 22 transfer RNA (tRNA) were predicted by tRNAscan-SE Search Server V.1.21 (Lowe et al. 1997) and ARWEN (online version) (Laslett and Canbäck 2007). The graphical map of the complete mitochondrial genome was drawn using the online software OrganellarGenomeDRAW (Lohse et al. 2007).

The complete mitogenome of *Gasterosteus aculeatus* (GenBank accession: MH205729) is a closed-circular molecule of 16,543 bp in length, which is a litter longer than *Gasterosteus wheatlandi* (16,538 bp). Like most other metazoan mitogenomes, it contains 13 PCGs, 22 tRNAs, two rRNAs and a large noncoding control region. Among the 37 genes, there are two rRNAs, 12 PCGs, and 14 tRNAs encoded in the J strand, while ND6 and the rest eight tRNAs are encoded in the N strand. The overall nucleotide composition is: 27.0% A, 28.4% T, 27.4% C, and 17.2% G, with a total G + C content of 44.6%.

To construct the phylogenetic relationship within Gasterosteales, other 16 complete mitogenomes of Gasterosteales were downloaded from GenBank. Among these mitogenomes, *Spinachia spinachia* (GenBank accession: AB445128.1) was used as the outgroup. The genome-wide alignment of all mt genomes was done by HomBlocks (Bi et al. 2018), resulting in 15,711 positions in total, including almost all whole or partial PCGs and rRNA genes. These concatenated sets were used to reconstruct the phylogenetic relationships by maximum likelihood (ML) methods in MEGA6.0 (Tamura et al. 2013). The ML analysis was performed using default parameters and the confidence values of the ML tree were evaluated via a bootstrap test with 1000 iterations. As shown in Figure 1, the phylogenetic positions of these 17 mt genomes were successfully resolved with almost all 100 bootstrap supports. By phylogenetic analysis using ML method, *G. aculeatus* showed the closest relationship with the blackspotted stickleback (*Gasterosteus wheatlandi*).

**Figure 1.**
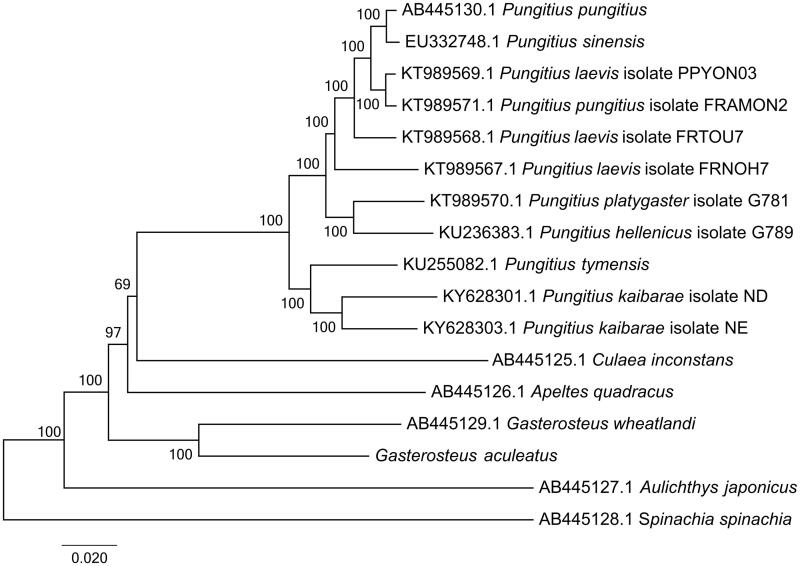
Phylogenetic relationships among 17 Gasterosteales mitochondrial genomes. The length of branch represents the divergence distance. Numbers beside nodes are percents of 1000 bootstrap values. GenBank accession numbers of mitochondrial genomes used in this phylogeny analysis were also listed.
